# A questionnaire-based survey of COVID-19 transmission in dental practice during the pandemic: comparison between the 1st–5th and the 6th–8th surges in Japan

**DOI:** 10.2340/aos.v84.43420

**Published:** 2025-05-13

**Authors:** Hironori Sakai, Eiji Kondo, Hirokazu Tanaka, Akinobu Shibata, Shizuka Nakatani, Hiroshi Kurita

**Affiliations:** Department of Dentistry and Oral Surgery, Shinshu University School of Medicine, Matsumoto, Japan

**Keywords:** COVID-19, dental care, infection control, communicable diseases, surveys and questionnaires

## Abstract

**Objectives:**

A previous questionnaire survey on infection control measures and infection status among practicing dentists during the 1st–5th surge of coronavirus disease 2019 (COVID-19) cases in Japan indicated a low risk of COVID-19 infection spreading through dental care. However, the low number of infected patients during the survey period may have been a contributing factor, and a sharp increase in the number of infected patients was subsequently observed. We re-examined the spread of infections in dental care settings and compared the results with those of previous reports.

**Materials and methods:**

An online questionnaire-based survey was administered in March 2023 to examine the situation from February 2022 to March 2023, when the 6th–8th surge of COVID-19 infection was observed in Japan. The survey was conducted via an online platform (Google Forms; San Mateo, California, USA). The call for participation was publicized to members of the Nagano Dental Association. The survey consisted of questions on clinical activities, infection control measures, and confirmed or probable COVID-19 cases among patients and clinical staff.

**Results:**

The number of COVID-19-positive patients increased approximately 50-fold between the study periods. There was a 3.5-fold increase in the rate of dental treatment for infected patients.

**Conclusion:**

Even with the increased likelihood of contact with COVID-19 patients, no cases of infection during dental treatment were observed. The results of this study indicate that even with the possibility of contact with COVID-19 during dental treatment, the likelihood of COVID-19 clusters occurring in dental practices is low if appropriate infection prevention measures are in place.

## Introduction

Severe acute respiratory syndrome coronavirus 2 (SARS-CoV-2) is a highly infectious coronavirus that emerged in December 2019 [1, 2]. SARS-CoV-2 has caused severe disease (coronavirus disease 2019 [COVID-19]) outcomes and has rapidly spread since the beginning of 2020 in Japan. The primary source of transmission of COVID-19 is person-to-person contact, and the routes of transmission of COVID-19 include direct transmission via coughing, sneezing, and inhalation of droplets, as well as contact transmission via oral, nasal, and ocular mucosal contact, droplets, and aerosols [3, 4]. SARS-CoV-2 has been detected in saliva samples, making saliva a potential transmission route for COVID-19. Healthcare workers in dental practice are at particular risk of SARS-CoV-2 infection due to close contact with patients and potential exposure to saliva-contaminated droplets and aerosols generated during dental procedures [5]. Therefore, dentists, dental personnel, and dental patients are potentially at high risk for exposure and transmission of this virus [6]. However, few studies have reported the occurrence of COVID-19 clusters in dental offices or dental departments of hospitals [7–10]. In addition, there is no clinical evidence that aerosols generated during dental procedures can lead to the transmission of COVID-19 [11].

In a previous study, we conducted a questionnaire survey on infection control measures and infection status among practicing dentists in Nagano Prefecture of Japan during the period from February 2020 to September 2021 (1st–5th surge of COVID-19 cases in Japan) to understand the status quo of infection in dental practices [12]. The results revealed that the risk of COVID-19 infection spreading through dental care was very low. It was suggested that COVID-19 clusters might not occur in dental settings where appropriate protective measures are implemented. However, the incidence of COVID-19 patients at the time of the survey (cumulative number of patients during the study period: 8,723; percentage of the population of the prefecture testing positive for COVID-19: approximately 0.43%) was not high, and the low incidence could be one of the reasons for the low risk of infection.

**Figure 1:**
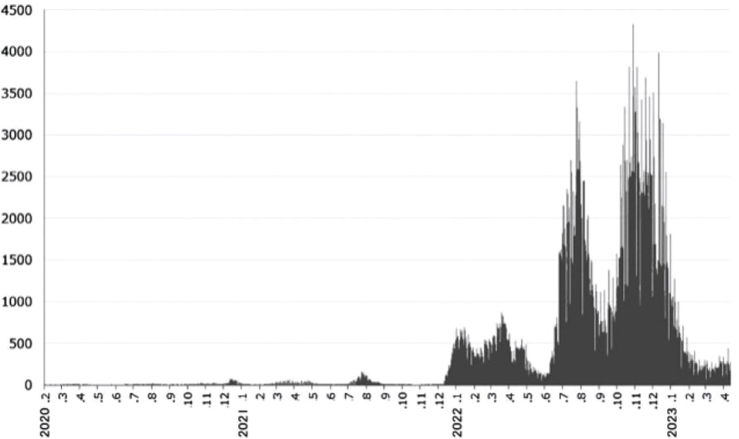
The population of COVID-19-positive patients in Nagano Prefecture.

A subsequent explosion in the number of infected patients (6th–8th surge of COVID-19 cases) and the emergence of new mutant viruses were confirmed in Japan. While the cumulative number of infected patients (polymerase chain reaction [PCR]-positive patients) from the 1st to 5th surge was 8,723, the number dramatically increased to 440,000 from the 6th to 8th surge, which means that approximately 21.8% of the population of Nagano Prefecture was COVID-19-positive (Figure 1). This also increased the possibility of nosocomial outbreaks in dental care settings. Therefore, in order to re-evaluate the situation of nosocomial infections in dental practice, a questionnaire survey was again conducted on the situation in which the number of infected patients had increased.

## Materials and methods

An online questionnaire survey was administered to members of the Nagano Dental Association in March 2023. The methods of the study were similar to those described in a previous report [12], wherein the data collection was based on an online platform (Google Forms; San Mateo, California, USA). The online questionnaire survey utilized the contact network of the Nagano Dental Association to invite members of the association to participate in the survey. The questionnaire consisted of questions on the number of patients, clinical activities (administrative control), infection control (vaccination, environmental/engineering control, and use of personal protective equipment [PPE]), and confirmed or suspected COVID-19 infection among patients and clinical staff. This study examined the situation from February 2022 to March 2023, when the 6th–8th surge of COVID-19 infection was observed in Japan. In Japan, active follow-up of coronary infections had been undertaken, and the diagnosis of infection was made in accordance with national guidelines. This study was conducted in accordance with the Declaration of Helsinki and was approved by the Medical Ethics Committee of Shinshu University (approval number 5306).

## Results

A total of 345 dentists responded to the survey (overall response rate: 31.0%). The results of the survey are summarized in Tables 1–4. Regarding treatment and patient restrictions (Table 1), 61.2% of respondents in the previous survey indicated that there were restrictions, while the percentage decreased to 37.7% in the current survey. There were no major changes in terms of what was restricted; however, there was a decrease in restrictions on procedures generating aerosols and droplets (17.9% to 8.5% and 12.3% to 3.8%, respectively). A comparison of infection control measures with the results of the previous survey is shown in Table 2. The vaccination rate (all staff and some staff members) was 97.1%, similar to that reported in the previous survey. Entrance screening for COVID-19 (symptoms/signs, body temperature, facemask use, and hand hygiene) was carried out in 93.3% of the clinics, a 3% decrease from the previous survey. Regarding infection control measures during procedures that generate splashes and/or aerosols, the results of this survey were similar to those of the previous survey. Surgical gloves (96.5%) and masks (surgical masks, 75.7%; N95 masks, 19.7%) were used in almost all clinics. Face/eye protection was used in approximately 80% of the clinics, and an extraoral dental suction device was used in approximately 75% of the clinics. The cleaning of potentially contaminated surfaces was performed in almost all clinics (92.8%), and draping was performed in 18.3% of the clinics. More than 80% of the clinics encouraged mouth washing before oral examination and treatment (24.1% with water and 58.0% with mouthwash).

**Table 1:** Clinical activities between February 2022 to March 2023.

Questions	Response	*n*		%	
Did you implement any restrictions on treatment and/or the patient?	Yes	130	235	37.7	61.2
	No	215	149	62.3	38.8
If yes, what type of restriction	Suspension of treatment	13	27	10.0	11.5
did you implement? (multiple answers allowed)	Restriction/coordination of patient number	119	194	91.5	82.6
	Stop accepting new patients	5	21	3.8	8.9
	Patient (disease) limitations (only urgent and/or critical patients)	21	53	16.2	22.6
	Restriction of aerosol-generating procedures (e.g. limit the number of procedures)	11	42	8.5	17.9
	Prohibition of procedures involving splashes	5	29	3.8	12.3
	Others	2	0	1.5	0
What was your policy for dealing with dental patients with suspected or confirmed COVID-19?	Postpone the treatment	202	220	58.6	57.3
	Provide palliative/emergency care	95	77	27.5	20.1
	Provide care with infection control measures	13	14	3.8	3.6
	Referral to hospital dentistry	17	36	4.9	9.4
	Others	18	7	5.2	1.8

Results in parentheses are for 2020/Feb.–2021/Sep.

Abbreviation: COVID-19: coronavirus disease 2019.

**Table 2:** Infection control measures between February 2022 to March 2023.

Questions	Response	*n*		%	
Vaccination status	All staff completed	278	(296)	80.6	(77.1)
	Some staff completed	57	(84)	16.5	(21.9)
	None completed	10	(4)	2.9	(1.0)
Did you perform any entrance screening?	Yes	322	(370)	93.3	(96.4)
	No	23	(14)	6.7	(3.6)
If yes, what type of screening did you perform? (multiple answers allowed)	Ask for symptoms/signs, close contact exposure, and travel from endemic areas of COVID-19	262	(334)	81.4	(87.0)
	Body temperature measurement	290	(343)	90.1	(89.3)
	Wear a mask	286	(346)	88.8	(90.1)
	Hand hygiene compliance	262	(331)	81.4	(86.2)
	Antigen test	5	(6)	1.6	(1.6)
What infection control measures were employed during the procedures generating splash and/or aerosol?	Use of surgical mask	261	(326)	75.7	(84.9)
	Use of N95 mask	68	(56)	19.7	(14.6)
	Use of surgical gloves	333	(377)	96.5	(98.2)
	Use of face/eye guard	276	(337)	80.0	(87.8)
	Use of surgical gown	39	(42)	11.3	(10.9)
	Use of surgical apron	108	(134)	31.3	(34.9)
	Use of cap	91	(106)	26.4	(27.6)
	Use of extraoral dental suction device	259	(305)	75.1	(79.4)
	Cleaning of possibly contaminated surface	320	(365)	92.8	(95.1)
	Cover possibly contaminated surface	63	(77)	18.3	(20.1)
	Mouth rinse with water	83	(100)	24.1	(26.0)
	Mouth rinse with mouthwash	200	(221)	58.0	(57.6)
	Installation of partition between units	168	(181)	48.7	(47.1)
	Installation of air purifiers	268	(289)	77.7	(75.0)
	Treatment in a negative pressure room	1	(2)	0.3	(0.5)

Results in parentheses are for 2020/Feb. – 2021/Sep.

Abbreviation: COVID-19, coronavirus disease 2019

A total of 17 (4.9%) clinics experienced dental treatment of confirmed COVID-19-positive patients, including root canal treatment, prosthetics, cutting and draining pus, tooth extraction, and oral care (Table 3). As a result, no cases of nosocomial viral infection associated with these procedures have been reported.

**Table 3:** Dental treatment for patients with confirmed COVID-19 between February 2022 and March 2023.

Questions	Response	*n*	%
Did you have any experience of dental treatment for confirmed COVID-19 patients?	Yes	17*(9)	4.9 (2.3)
	No	328 (375)	95.1 (97.7)
If yes, what procedure was performed? (multiple answers allowed)	Root canal treatment	4	
	Medication	3	
	Prosthetics (fitting and abutment tooth preparation)	2	
	Incision and drainage	2	
	Tooth extraction	1	
	Oral care	1	
	Details unknown	4	
Were there confirmed cases of viral transmission from the patient to dental staff?	Yes	0	
	No	17	

Results in parentheses are for 2020/Feb.–2021/Sep.

* One before vaccination and 16 after vaccination. Abbreviation: COVID-19, coronavirus disease 2019.

A total of 62 (18.0%) clinics experienced dental treatment for patients who were later found to be infected (i.e. some staff members were in close contact with COVID-19 patients) (Table 4). The dental procedures performed on the patients included dental scaling, the restoration of tooth caries, denture fitting/adjustment, root canal treatment, and tooth extraction which could have resulted in droplet formation during the period of possible viral infection. However, as in the previous study, no cases of viral infection from patients to dental staff members were reported.

**Table 4:** Close contact exposure to COVID-19-positive patients between February 2022 and March 2023.

Questions	Response	*n*	%
Did you have any experience of treating patients with close contact exposure to COVID-19-positive patients?	Yes	62* (18)	18.0 (4.7)
	No	283 (366)	82.0 (95.3)
If yes, please describe how many days there were between treatment and confirmation of the patient’s COVID-19 infection	Within 3 days	39	
	4–7 days	16	
	8–14 days	2	
	Unknown	5	
If yes, what procedure was performed? (multiple answers allowed)	Dental scaling	16	
	Restoration of tooth caries	13	
	Denture fitting/adjustment	6	
	Root canal treatment	5	
	Tooth extraction	4	
	Attach dental restorations	2	
	Orthodontic treatment	2	
	Local cleaning	1	
	Mandible fracture	1	
	Others	8	
	No description	4	
Were there confirmed cases of viral transmission from the patient to the dental staff?	Yes	0	
	No	62	

Results in parentheses are for 2020/Feb.–2021/Sep.

* Four before vaccination and 58 after vaccination. Abbreviation: COVID-19, coronavirus disease 2019.

A total of 183 dentists reported that staff members, including the dentists themselves, had been infected with COVID-19 (20 before vaccination and 163 after vaccination). The source of infection was suspected to be outside the clinic in 106 and was unknown in the remaining occurrences. Of these, 15 (4%) indicated that there was infection among the clinical staff. Twenty-one of these staff members provided dental care during the period of possible viral infection, but none of the patients who received dental care were diagnosed with COVID-19 infection. Sixty (17.4%) reported that they had closed their clinic due to infection or close contact with COVID-19 patients.

## Discussion

In the present study, a similar questionnaire was administered to almost the same subjects to investigate COVID-19 transmission in dental practice during different infectious disease pandemics (1st–5th vs. 6th–9th surge in Japan). The results of this survey showed that no nosocomial infections were observed in the dental clinics even when the number of COVID-19 patients increased explosively. The number of COVID-19-positive patients increased approximately 50-fold between the study periods that were compared. In addition, the results of this study also showed that the number of COVID-19 patients treated at dental clinics also increased approximately 2-fold (from 9 to 17), and the number of dental staff identified as concentrated contacts of COVID-19 patients increased approximately 3.5-fold (from 18 to 62). Although dental care restrictions were in place in the early stages of the spread of the infection, the rate of dental care restrictions also declined, as indicated by the results of this survey (61.2% in the 1st–5th surge vs. 37.7% in the 6th–8th surge). As mentioned above, even with the increased likelihood of contact with COVID-19 patients, no cases of infection during dental treatment have been observed.

The results of a previous study [12] suggested that entrance screening (water-front measures), standard infection control measures such as contact infection control and standard PPE (masks, face shields, eye guards, gloves, gowns, and aprons), the use of extraoral vacuums, and encouraged gargling play important roles in infection control in dental practice. In the current survey results, these infection control measures were still being implemented at the same high rates as before. In addition, vaccination coverage was also high at approximately 80% in both study periods. These results suggest that the situation in which such infection control measures are implemented in dental practices is likely the reason why COVID-19 infections associated with dental treatment are virtually nonexistent.

In a 2009 report on infection control in Japan by Tada et al. [13], the use of masks and gloves was already very high. However, the use of extraoral vacuums was less than 30%, which was stated to be due to economic reasons. Moreover, it has already been recognized that extra-oral vacuums are one of the environmental measures used to prevent nosocomial infections. In addition, older dentists were less likely to have received infection control education; therefore, standard infection control measures were not sufficiently implemented.

One key difference between the present study and the aforementioned report by Tada et al [13] is the higher rate of use of extraoral vacuums in our study. In Japan, Subsidies for Dental Outpatient Facilities [14] was introduced in 2008 to introduce reimbursement for medical safety and infection control measures. The installation of a dental suction device is a mandatory requirement for the eligibility of this subsidy. There were 67,779 dental clinics in 2008, of which 2,868 (4.2%) had submitted applications for reimbursement [15]. This had increased in 2018, the year before COVID-19, with 68,613 clinics and 23,048 applications for reimbursement (33.6%) [16]. During the COVID-19 epidemic, the number of applications for reimbursement increased and surpassed the majority of clinics, as there were 67,281 clinics in 2023 of which 34,075 had applied for the subsidy (50.6%) [17]. Although the increased use of dental aspirators may not be entirely due to the introduction of government subsidies, it is possible that the introduction of insurance coverage for the device via reimbursement may have lowered the hurdle to developing and improving clinical facilities and equipment. In addition, the recent increase in student education and training opportunities for infection control may have increased the awareness of healthcare professionals, which may have contributed to the results of this study.

During the period of this study, a total of 183 dentists reported that staff members, including dentists themselves, had been infected with COVID-19. The results of this study suggested that transmission occurred outside dental care, at home, at school, at the office, or at other crowded indoor settings. In fact, in this survey, 15 indicated that there was COVID-19 transmission among clinical staff members outside dental care. The risk of viral transmission is high during unmasked communication [18, 19]. Infection of medical staff affects the delivery of healthcare [20]. At the time of this survey, 17.4% of dental clinics reported having to close their offices. In a pandemic of respiratory infections, the health of medical staff is important, as is attention to nosocomial infections.

As noted in our previous report, this survey has several limitations. First, this survey was conducted in a single prefecture in Japan. It is unclear whether similar results would be obtained in a larger national survey. Second, the overall response rate was 31.0%, which may indicate some degree of potential selection bias. Respondents may have been more concerned or worried about safety measures than non-respondents were. However, given the need for timely information and the rarity of medium to large surveys of COVID-19 measures in private dental practices, the data from this study may be useful to clinicians. Finally, because the survey was self-reported, there is a small possibility of misclassification by the individual practicing dentists who responded.

In conclusion, the results of this study indicate that even if COVID-19 infection was to spread and the number of infected patients was to increase approximately 50-fold in a short period of time, the likelihood of COVID-19 clusters occurring in dental practices is low if appropriate infection prevention measures are in place.

## Geolocation information

Japan

## Data Availability

All of the data supporting underlying findings are included in the manuscript.
